# Succinate dehydrogenase inhibitor seed treatments positively affect the physiological condition of maize under drought stress

**DOI:** 10.3389/fpls.2022.984248

**Published:** 2022-08-30

**Authors:** Dominika Radzikowska, Przemysław Łukasz Kowalczewski, Monika Grzanka, Romana Głowicka-Wołoszyn, Marcin Nowicki, Zuzanna Sawinska

**Affiliations:** ^1^Department of Agronomy, Poznań University of Life Sciences, Poznań, Poland; ^2^Department of Food Technology of Plant Origin, Poznań University of Life Sciences, Poznań, Poland; ^3^Department of Finance and Accounting, Poznań University of Life Sciences, Poznań, Poland; ^4^Department of Entomology and Plant Pathology, Institute of Agriculture, University of Tennessee, Knoxville, TN, United States

**Keywords:** abiotic stress, chlorophyll fluorescence, photosynthesis, SDHI, *Zea mays*

## Abstract

Improvements in agricultural production are needed, as the growing human population demands more resources and exerts stronger effects on climate. Water scarcity is one of the main factors limiting the yield of maize in many regions of the world. One possible method to mitigate the negative effects of drought is seed mortars; its use improves plant development from the early stages onwards. In this study, we tested 12 various seed treatments with and without succinate dehydrogenase inhibitors (SDHI; sedaxane) on maize “SY Fanatic.” Physiological parameters of germinating seeds, of young maize seedlings under drought, and of seedlings recuperated from drought were assessed and compared across 12 seed treatments and with non-stressed plants. The seed treatments varied greatly in their influence on the germination and the physiological state of seedlings under drought and after regeneration. Seeds under treatments No. 6, 11, and 12 showed the highest germination energy (97.3%). The use of SDHI-containing seed treatments significantly improved the development of the maize root system. The longest roots, ~13 cm in length, were recorded for treatments No. 6 and 12, both containing sedaxane. These treatments also boosted the functioning of plants growing under optimal soil moisture conditions and under drought stress, influencing the photosynthesis process, increasing the absorption of CO_2_, and improving the parameters of chlorophyll fluorescence in relation to non-treated controls. Our data indicated that using substances from the SDHI group can possibly reduce the drought-related stress reactions in maize, helping this important crop to face the progressing climate change.

## Introduction

Major global crops in high-yielding, temperate regions are suffering the increasing yield losses due to climate change, in particular from drought and heat, which often occur during the critical stages of plant vegetation (Snowdon et al., 2021). A distinction is made between regions that show temporary fluctuations in rainfall and those with permanent deficits (Wilhite and Glantz, 2019). Meteorological drought refers to a period with no precipitation affecting the entire region. Agricultural drought can be defined as a state where the evapotranspiration demand exceeds the amount of water that can be taken by the cultivated plant. More broadly, the drought stress can be defined as the point at which the water content of the soil acts as a limiting factor in plant transpiration (Gosa et al., 2019). The total annual rainfall does not necessarily change significantly under the ongoing climate change, but the frequency and distribution of rainfall may. For the phenologically adapted crops, this may result in the timing of water supply adversely related to the needs of the plant, for example by shifting toward off-season precipitation (Bönecke et al., 2020). Moreover, even a slight increase in spring temperatures can lead to earlier and faster growth of crops, causing them to use more water in the early stages of development and to potentially run out of water, e.g., in early summer (Lian et al., 2020).

Due to its wide variety of uses, maize (*Zea mays* L.) is one of the most important crops in the world. Maize seeds are used both for food and feed, but the plant is also of industrial importance, for example in the production of bioenergy, and as a raw material for other industries. It is a multifunctional plant with wide adaptability to various agro-climatic conditions. Maize is widely cultivated in most regions of the world, at altitudes of up to 3,000 m above sea level, owing to the highest grain production potential among cereals (Chakraborty et al., 2012; Madhav et al., 2016; Sah et al., 2020). In many countries, maize is grown in areas with the annual rainfall levels of 300 to 500 mm, although that amount is below the critical level for good yields (Monfreda et al., 2008; Hanjra and Qureshi, 2010).

The use of seed treatments is the most popular way of applying fungicides and insecticides due to the very high efficiency of protection for the future plant. Adequate protection of maize is of great importance as the plant is frequently exposed to biotic and abiotic stresses. The effects of seed fungicides on plants vary according to the growing conditions. Under low pathogen pressure, such protection does not improve the emergence nor yield of grain, but under high pressure, it certainly increases the benefits of such application (Sierotzki and Scalliet, 2013; Baibakova et al., 2019).

For the plant protection compounds-pesticides, fertilizers, bio-stimulants-to take effect on the seedlings during germination or at the seed-soil interface immediately after sowing, they are routinely applied on the surface of the seeds, i.e., seeds are coated. The use of seed treatments allows to reduce the impacts of pathogens, which in turn improves the condition of plants from the earliest stages of development onwards (Montfort et al., 1996; Sawinska, 2008; Sawinska et al., 2014). Fungicides, including seed treatments, began to be used as early as 1,600 (Deliopoulos et al., 2010). The most important group of fungicides used in seed treatment are the methionine demethylation inhibitors, which affect the biosynthesis of sterols—triazoles, imidazoles, pyrimidines—in fungi (Kuck et al., 1995). Among many modern fungicide preparations for seed treatment, a combination of several active ingredients with various modes of action-systemic and contact-can be found. Such mixtures show a broad spectrum of the pathogens being controlled and also reduce the likelihood of resistance selection (Ayesha et al., 2021; Jørgensen and Heick, 2021).

Succinate dehydrogenase inhibitors (SDHI) inhibit the succinate dehydrogenase complex in the complex II of pathogen respiratory chain (Kuhn, 1984; Stammler et al., 2007; Avenot et al., 2008). The currently used SDHI formulations show a broad spectrum of activity against many fungal pathogens in crops (Avenot and Michallides, 2007; Sierotzki and Scalliet, 2013). In addition to protection, SDHI can also enhance rooting and may also be beneficial in overcoming the biotic and abiotic stresses in the early stages of growth (Sierotzki and Scalliet, 2013; Dal Cortivo et al., 2017; Radzikowska et al., 2020).

In addition to pathogens, also the abiotic stress factors can limit plant growth; the most-studied abiotic stresses include water shortages, high temperatures, and low humidity, all of which led to drought stress (Mittler, 2006; Bandurska et al., 2017; Swędrzyńska et al., 2019). Abiotic stress is perceived as the main factor influencing the crop yield reduction; it can reduce the average yields by up to 50% (Li et al., 2009). Water shortage during the growing season of plants can trigger numerous problems in basic physiological and metabolic reactions. It can also cause an increased production of reactive oxygen species, which additionally adversely affects the condition of plants (You and Chan, 2015). Plants have developed various protective mechanisms against the action of reactive oxygen species, which include the stimulation of effective enzymatic and non-enzymatic antioxidant defenses (Ghorbanpour et al., 2020). Nevertheless, in the face of global climate change, including the increases in average temperature and the fluctuations in rainfall, methods to increase the tolerance of plants to abiotic stress factors are needed. Analyses of gene expression, chlorophyll gas exchange, and fluorescence measurements data convergently showed that SDHI fungicides increase the photosynthesis intensity and the efficiency of photosystem II (PSII) in plants under drought stress (Ajigboye et al., 2017; Radzikowska et al., 2020).

Both drought stress and pathogen infections substantially affect the physiological condition of plants. By eliminating or even reducing abiotic or biotic stresses, it is possible to mitigate yield losses; therefore, the aim of this study was to test the hypothesis of SDHI benefits on maize seed and seedling physiology under abiotic stress. We did this using by testing various formulations of seed treatments with or without SDHI, and by assessing the physiological state of plants and their reactions to drought stress.

## Materials and methods

### Plant material and quality assessment of maize seeds

Maize (*Zea mays* L.) cultivar “SY Fanatic” (Syngenta) was used as the model plant in our study. The estimation of seed sowing quality was carried out in accordance with the protocols from Main Inspectorate of Plant Health and Seed Inspection of Poland and the International Seed Testing Association (Dąbrowska et al., 2000; ISTA, 2011). This included assessments of germination energy, germination capacity, and vigor index. Furthermore, the root length and the shoot length were determined. The seedling growth test was performed on a roll test basis, on a 25-seed sample, in four replications. Each roll consisted of 3 layers of wetted filter paper 30 cm × 45 cm (quality paper type R with retention time of 30 s). The rolls were placed in a thermostatic cabinet “ST 5+” (Pol-Eko-Aparatura, Wodzisław Śląski, Poland) set at a temperature of 19°C. The maize germination energy was determined after 4 days, and the germination capacity after 7 days. After this time, the length of normally developed seedlings was measured. The vigor index (VI) was calculated according to the equation (1):


(1) $$VI=\mathrm{seedling length}\;\left(\mathrm{cm}\right)\times \mathrm{germination}\;\left(\%\right)$$

Twelve various seed treatments were used before sowing (Table 1). The control group consisted of the non-treated seeds. The concentrations of the substances were selected in accordance with the manufacturers’ recommendations used for the agricultural practice.

**Table 1:** Active substances and trade names of seed treatments used in experiment.

No.	Active substances of seed treatments	Trade names of seed treatments
1	Control – untreated	
2	fludioxonil 2.40% + metalaxyl-M 0.93%	Maxim^®^ XL 034,7 FS
3	fludioxonil 2.40% + metalaxyl-M 0.93% + sedaxane 42.8%	Maxim^®^ XL 034,7 FS + Vibrance^®^ 500 FS
4	fludioxonil 2.40% + metalaxyl-M 0.93% + sedaxane 42.8% + tefluthrin 200 g/L	Maxim^®^ XL 034,7 FS + Vibrance^®^ 500 FS + Force^®^ 20 CS
5	fludioxonil 3.32% + metalaxyl-M 2.65% + azoxystrobin 1.33% + thiabendazole 26.50%	Maxim Quattro
6	fludioxonil 3.32% + metalaxyl-M 2.65% + azoxystrobin 1.33% + thiabendazole 26.50% + sedaxane 42.8%	Maxim Quattro + Vibrance^®^ 500 FS
7	fludioxonil 3.32% + metalaxyl-M 2.65% + azoxystrobin 1.33% + thiabendazole 26.50% + sedaxane 42.8% + tefluthrin 200 g/L	Maxim Quattro + Vibrance^®^ 500 FS + Force^®^ 20 CS
8	sedaxane 42.8%	Vibrance^®^ 500 FS
9	tefluthrin 200 g/L	Force^®^ 20 CS
10	sedaxane 42.8% + tefluthrin 200 g/L	Vibrance^®^ 500 FS + Force^®^ 20 CS
11	metalaxyl 1.87% + prothioconazole 9.35%	Redigo^®^ M 120 FS
12	metalaxyl 1.87% + prothioconazole 9.35% + sedaxane 42.8%	Redigo^®^ M 120 FS + Vibrance^®^ 500 FS
13	metalaxyl 1.87% + prothioconazole 9.35% + sedaxane 42.8% + tefluthrin 200 g/L	Redigo^®^ M 120 FS + Vibrance^®^ 500 FS + Force^®^ 20 CS

### Rhizobox study

To separate the root zone from the above-ground parts of plants, the rhizobox chambers were used; this allowed to visually assess the development of the root system in the plants. Completely transparent rhizoboxes were made of PVC boards glued together with silicone sealant. Their internal dimensions were: 17 × 17 × 1 cm (h × w × d). Each of the rhizoboxes was filled with a layer of peat up to 8 cm high, on which 5 maize seeds were placed. These were then covered with a 2 cm high layer of sand and closed. The soil moisture content was kept constant throughout the experiment by adding water daily *via* a pipette to keep the weight of the rhizobox constant. The rhizoboxes were placed in the greenhouse under conditions identical to those of the potted plants. Three rhizobox replicates were analyzed for each seed treatment combination (1 through 13; Table 1).

### Plant growing conditions

Plants were grown for 36 days in potted cultures in a greenhouse (60% to 80% relative humidity, 20 to 25°C, 16 h day/8 h night). Plants were grown under natural sunlight supplemented with 400 W sodium lamps (HPS; Elektro-Valo Oy Netafim, Avi: 13473, Uusikaupunki, Finland). Five maize seeds of equal size were sown in triplicate, in each 1.0 dm^3^ pot filled with the same amount of soil substrate (universal substrate, pH 5.5). Before the measurements, the plants were thinned until 3 per pot. Soil moisture was maintained at a constant level of 20% to 22% v/v by watering every 48 h (100 ml H_2_O/pot). Soil moisture was monitored every day with a ThetaProbe (Eijkelkamp, Netherlands). Drought stress was imposed by stopping to water at the sixth leaf stage, and 5 days thereafter the signs of drought manifested as loss of turgor and saber-like leaf curl. At that time, the soil moisture reached 6% to 8% v/v and became hardly available to plants. At that stage, the physiological state of plants was determined in both the watered control and the drought-stressed plants. At 9 h before the measurements, the plants were placed in darkness to repress the photosynthesis. The measurements were carried out in a phytotron at a constant air temperature of 25°C and 70% ± 5% relative humidity. After measurements, the plants were transferred back to the greenhouse and watered to bring the soil moisture to 22% v/v. After 6 days, when all the plants regained the turgor, second measurement of their physiological state was taken. Measurements of the physiological state of plants during drought stress as well as after turgor regeneration were carried out in the same way. Also, the order of measurements was maintained the same: drought-stressed plants and watered plants (controls) were measured alternately for each respective seed treatment. For each plant undergoing assessment, the youngest, fully developed leaf was analyzed for the photosynthesis and chlorophyll fluorescence measurements.

### Physiological state of maize plants

#### Relative water content

Plant water status was estimated by measuring leaf RWC of control and drought-stressed plants. Two-centimeter slices of freshly collected leaf from control and drought-stressed plants were weighed three times in weighing dishes. The first weighing was carried out after collecting the plant material (fresh mass; f.m.), the second one after soaking the plant material for 4 h in distilled water (f.m. in full turgor), and the third weighing after drying the plant material for 4 h in 70°C (dry mass; d.m.). RWC measurements were performed in three replications. RWC was calculated using the formula: (2)


(2) $$RWC\;\left[\%\right]=\frac{f.m.-d.m.}{f.m.\mathrm{in full turgor}-d.m\text{.}}$$

where: f. m. – fresh mass, d. m. – dry mass.

#### Plant photosynthesis

The plant photosynthesis intensity values were measured twice – after drought stress imposition and after regeneration. Photosynthesis intensity was estimated on single leaves in the leaf chamber, based on CO_2_ exchange rate—A (mol/m^2^ × s), transpiration rate—E (mmol/m^2^ × s), sub-stomatal CO_2_—Ci (μmol/mol), and stomatal conductance—Gs (mol/m^2^ × s). The measurements were taken using a portable photosynthesis system (LCpro-SD, ADC BioScientific Ltd., Hoddesdon, United Kingdom) with a narrow leaf chamber (area: 5.8 cm^2^) on the first young fully developed healthy leaf. Photosynthesis measurements were carried out in triplicate (three plants) for each seed treatment at either timepoint. The CO_2_ concentration (reference CO_2_) in the leaf chamber was kept at 360 vpm, leaf chamber temperature (Tch) was set at 25°C, the flow rate of air (u) was kept at 200 μmol/s, and ambient H_2_O concentration (Reference H_2_O) was used. Photosynthetically active radiation (PAR) was kept at 400 μmol/s × m^2^, adjusted automatically by a red-blue light-emitting diode light source (LCP Narrow Lamp, ADC BioScientific Ltd.).

#### Plant chlorophyll fluorescence

Chlorophyll fluorescence was measured for the same leaf and at the same light intensity as photosynthesis at PAR = 400 μmol/s × m^2^, using Multi-Mode Chlorophyll Fluorometer (OS5p, Opti-Sciences, Inc., Hudson, NY, United States) with a PAR Clip that allows measuring both PAR and leaf temperature. A kinetic test mode was used; it combines the measurements under the light conditions and the measurements after the adaptation to darkness. Fluorescence measurements were carried out in six replicates for each combination. The fluorometer settings protocol followed our previous studies (Kowalczewski et al., 2020; Radzikowska et al., 2020): Modulation Source: Red, Modulation Intensity: 29, Detector Gain: 06, Saturation Flash Intensity: 30, Flash Count: 001, Flash Rate: 255 (sec). The following parameters were measured after the adaptation to darkness: F0 – minimum fluorescence, Fm – maximum fluorescence, Fv/Fm – maximum quantum efficiency of PSII photochemistry, and under the light conditions: Y – quantum yield of photosynthetic energy, ETR – electron transport rate.

### Statistical analysis

The effect of two factors (presence of drought stress and type of seed treatment) on the physiological state of plants was examined using two-way ANOVA and Tukey’s HSD algorithm at *α* = 0.05 with three independent replicates (six for the plant chlorophyll fluorescence parameters) using Statistica 13.3 (Dell Software Inc., Round Rock, TX, United States), according to the method described previously (Radzikowska et al., 2020) and R v4.1.2 using package agricolae v1.3–5. The impact of applied treatments on germination energy, germination capacity, vigor index, root and shoot length of maize seeds, as well as the rhizobox study were examined using one-way ANOVA and Tukey’s HSD algorithm at *α* = 0.05 with three independent replicates using Statistica and R with package agricolae. R v4.1 with packages FactoMineR v2.4 and factoextra v1.0.7 (Michael, 2010) were used for principal components analysis (PCA) to illustrate the results obtained.

## Results and discussion

### Effect of seed treatment on seed quality, germination energy, and germination capacity

Prompted by findings of root stimulation in wheat (Barchietto et al., 2012), we investigated the side-effects of sedaxane in maize over and above its protective capacity, in addition to other seed treatments. We found that seed treatment significantly affected the morphological traits of maize roots (Table 2). Our results indicated that the germination energy of the seeds varied significantly depending on the treatment applied and ranged from 85.3% for treatment No. 4 to 97.3% for treatments No. 6, 11 and 12. On the other hand, no significant influence of the seed treatment on germination capacity was noted (Table 2). Application of the seed treatments also significantly affected the vigor index, and seeds under treatment No. 6 had the highest values (586.88). The lowest value of this index was noted for seeds under treatment No. 7 (447.81). Analysis of the average length of maize sprouts showed that the same treatment No. 7 reduced the length of the seedlings the most among all the analyzed formulations. The longest shoot length, 6.3 cm in average, was observed for treatment No. 6, whereas the longest roots were observed for treatments No. 6 at 13.31 cm and No. 12 at 13.17 cm (Table 2). This is consistent with results reported by Colla et al. (2014) on maize coleoptile elongation with protein hydrolysates. Such root morphology responses were also observed to other bio-stimulating compounds (Calvo et al., 2014).

**Table 2:** Impact of applied treatments on germination energy, germination capacity, vigor index, root and shoot length of maize seeds.

Treatment^*^	Germination energy (%)	Germination capacity (%)	Shoot length (cm)	Root length (cm)	Vigor index
1	96.0 ^ab^	97.3 ^a^	5.53 ^abc^	11.88 ^abc^	537.63 ^ab^
2	89.3 ^bc^	94.7 ^a^	5.84 ^ab^	12.39 ^ab^	554.67 ^ab^
3	96.0 ^ab^	97.3 ^a^	5.71 ^abc^	12.30 ^ab^	556.16 ^ab^
4	85.3 ^c^	94.7 ^a^	5.24 ^bcd^	10.80 ^bc^	498.11 ^abc^
5	94.7 ^ab^	96.0 ^a^	5.87 ^ab^	12.48 ^ab^	566.45 ^ab^
6	97.3 ^a^	97.3 ^a^	6.03 ^a^	13.31 ^a^	586.88 ^a^
7	89.3 ^bc^	94.7 ^a^	4.72 ^d^	11.05 ^bc^	447.81 ^c^
8	92.0 ^abc^	98.7 ^a^	5.07 ^cd^	11.75 ^abc^	499.60 ^abc^
9	94.7 ^ab^	97.3 ^a^	5.71 ^abc^	11.82 ^abc^	555.33 ^ab^
10	90.7 ^abc^	96.0 ^a^	5.11 ^cd^	10.23 ^c^	490.24 ^bc^
11	97.3 ^a^	98.7 ^a^	5.16 ^cd^	11.72 ^abc^	508.93 ^abc^
12	97.3 ^a^	100 ^a^	5.57 ^abc^	13.17 ^a^	557.33 ^ab^
13	96.0 ^ab^	96.0 ^a^	5.05 ^cd^	12.72 ^ab^	485.23 ^bc^
LSD	6.71	6.45	0.677	1.955	84.401

Means of indices for four independent seedlings are shown. ^a–d^Different letters in columns indicate statistically different mean values *α* = 0.05.

* 1–13 numbers indicate active substances of seed treatments used, according to Table 1.

### Rhizobox study

**Figure 1:**
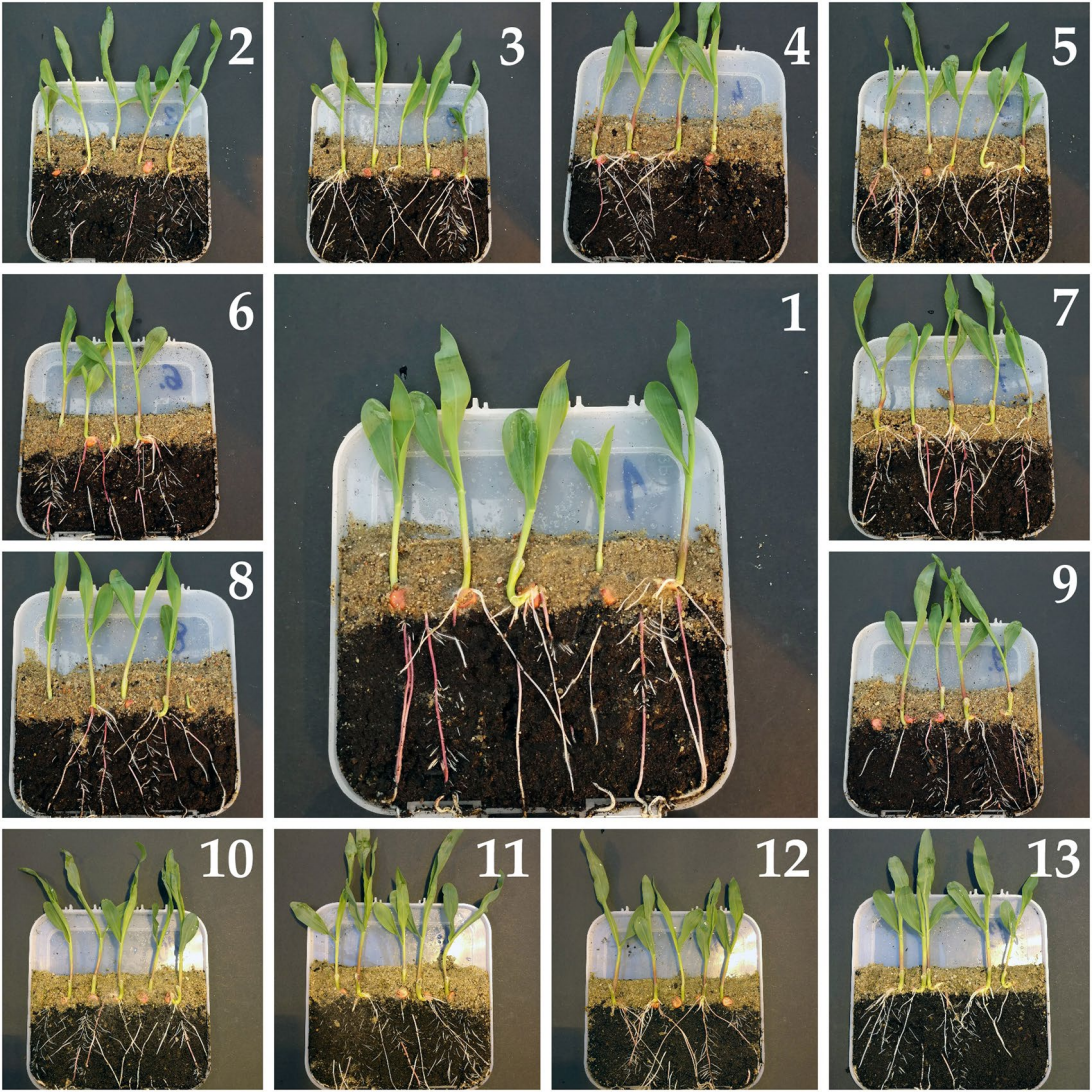
Rhizobox test allows to track root and seedling development as affected by various seed treatments. 1–13: Composition of seed treatments as shown in Table 1. Pictures show maize seedlings grown for 21 days.

The use of rhizoboxes allows for a thorough analysis of the plant root system. The box inclination during cultivation forces the root system to grow along a flat, transparent wall, which allows to fully track the growth of the root system and facilitates its analysis (Mašková and Klimeš, 2020). Roots are notoriously difficult to study. Soil is a visual and a mechanical barrier alike, making it difficult to track roots *in situ* without destructive harvest or expensive equipment. Visualizing and measuring root growth *in situ* is extremely challenging (Schmidt et al., 2018). The use of rhizoboxes allowed us for the non-destructive visualization of maize root growth and for the assessment of the influence of various seed treatments. We observed differences in root plasticity in response to various seed coatings (Figure 1). Better-developed root system is clearly visible for seed treatments containing the SDHI (Figure 1; Table 2). For those treatments, visibly higher numbers of fine roots were developed, indicating a superior effect of sedaxane on the root morphology (Figure 1). Previous studies showed that sedaxane exerted significant auxin-like and gibberellin-like effects, with marked morphological and physiological changes according to an approximate saturation dose–response model (Dal Cortivo et al., 2017). In addition to its protective effect, sedaxane can facilitate root establishment and intensify nitrogen and phenylpropanoid metabolism in young maize plants and aid in overcoming biotic and abiotic stresses at the early growth stages (Dal Cortivo et al., 2017).

### Effect of the seed treatments on the physiological state of plants under drought stress

A leaf relative water content (RWC) is a measure of its hydration state relative to its maximum water holding capacity at full turgor (González and González-Vilar, 2001). The applied seed treatments exerted significant impacts on the RWC, in both control and drought-stressed maize seedlings. The demonstrated interaction of the treatments applied with the stress response arose because although in all combinations there was a significant decrease in the RWC value under drought, those decreases varied. Overall, we observed a significant effect of drought imposition on the RWC, as well as the effects of the seed treatment and interaction of the factors (all at *p* < 0.001). RWC values in drought-stressed combinations ranged between 57% for untreated control and 73% for plants whose seeds were treated with treatment No. 12. The significantly lowest RWC among treatments was found for treatment No. 2 (Table 3; Supplementary Table 1).

**Table 3:** The influence of the seed treatment and drought stress on relative water content – RWC (%) during drought stress.

Treatment^*^	Presence of drought stress^2^		Mean^1^
	Control	Drought	
1	83.85 ^e^	57.05 ^k^	70.45 ^a^
2	84.38 ^de^	59.97 ^j^	72.18 ^a^
3	86.29 ^bc^	68.83 ^g^	77.56 ^a^
4	89.73 ^a^	69.62 ^g^	79.67 ^a^
5	85.81 ^cd^	65.32 ^h^	75.56 ^a^
6	86.21 ^bc^	69.46 ^g^	77.84 ^a^
7	87.77 ^b^	70.35 ^g^	79.06 ^a^
8	85.87 ^cd^	66.02 ^h^	75.94 ^a^
9	85.27 ^cde^	63.50 ^i^	74.38 ^a^
10	85.61 ^cde^	65.39 ^h^	75.50 ^a^
11	84.53 ^cde^	63.23 ^i^	73.88 ^a^
12	90.07 ^a^	72.89 ^f^	81.48 ^a^
13	84.62 ^cde^	62.38 ^i^	73.50 ^a^
**Mean** ^**1**^	86.15 ^a^	65.69 ^b^	
**LSD**_**Stress**_	1.536		
**LSD**_**Treatment**_	22.485		
**LSD**_Stress*Treatment_	1.793		

Means of indices for three independent seedlings are shown. ^a–k^Different letters indicate statistically different mean values of Tukey’s HSD at *α* = 0.05.

1 One-or.

2 Two-Way ANOVA was used for the means separation. LSD denote the Least Significant Difference values calculated using the same method, either for single factors (Treatment; Stress) or their interaction.

* 1–13 numbers indicate active substances of seed treatments used, according to Table 1.

Pietragalla and Mullan (2012) argued that plant genotypes with the ability to maintain the full leaf turgor under drought overcome the effects of that stress. This allows for the proper course of turgor-dependent processes, such as plant growth, activity of the stomata, and of PSI and PSII (Pietragalla and Mullan, 2012). Therefore, the objects with the highest RWC under drought stress will be able to better cope with stress at many physiological levels. Maize seeds priming with Se + Zn increased RWC during drought by 27%, whereas individual Se or Zn seed treatments improved the RWC by 16% and 11%, respectively (Nawaz et al., 2021). Plant hormones play a crucial role in the defense responses to abiotic stresses, and exogenous applications of specific growth regulators increase the tolerance of plants to drought stresses (Madhava Rao et al., 2006). Exogenous applications of methyl jasmonate or salicylic acid increased the RWC by 43% or 272%, respectively, from unstressed to drought-stressed conditions (Tayyab et al., 2020). Whereas, in the current study, the application of treatment No. 12 increased the RWC in unstressed plants by 7%, and in drought-stressed by 28%. Sedaxane, a fungicide functioning as inhibitor of succinate dehydrogenase, triggers in plants many physiological reactions related to the shifts of hormonal balance and changes in expression of numerous genes (Shkliarevskyi et al., 2019).

The assessment of photosynthetic activity was carried out on the basis of gas exchange measurements: CO_2_ assimilation (A) and H_2_O transpiration (E). In addition, the stomata conductance (Gs) and the intercellular CO_2_ concentration (Ci) were measured. Both during drought and after regeneration, significant effects based on One- and Two-Way ANOVA were attributed to the imposed stress, seed treatments, and the combination of factors, respectively. The significant influence (*p* < 0.001) of the applied seed treatments, of the stress imposition, and of the combination of both factors on the efficiency of photosynthesis measured during drought stress were observed (Table 4). The use of all seed treatments significantly increased the assimilation of CO_2_ (A) in control plants growing under optimal hydration conditions in relation to plants whose seeds were not treated, but under water stress, no significant increase in CO_2_ (A) assimilation was found in plants whose seeds were treated using treatments No. 9 and 13. The highest levels of CO_2_ assimilation under optimal irrigation conditions were observed for plants whose seeds were treated using treatments No. 12, 7, 6, 4, and 3, for which the increase in parameter A compared to the untreated control was 49%, 48%, 45%, and 44%, respectively. On the other hand, the measurement of photosynthesis under drought stress showed the highest CO_2_ assimilation in plants treated with treatments No. 12, followed by 4, 3, and 7, and it increased in relation to plants with untreated seeds by 439%, 336%, 304%, and 239%, respectively (Table 4).

**Table 4:** The influence of the seed treatment and drought stress on CO_2_ assimilation – A (μmol/m^2^ × s) during drought stress and after regeneration.

Treatment^*^	During drought stress^2^			After regeneration^2^		
	Control	Drought	Mean^1^	Control	Drought	Mean^1^
1	10.44 ^ef^	2.09 ^kl^	6.27 ^a^	7.40 ^l^	11.16 ^k^	9.28 ^f^
2	11.74 ^de^	1.41 ^l^	6.58 ^a^	11.73 ^jk^	12.04 ^ijk^	11.89 ^e^
3	14.94 ^a^	8.44 ^gh^	11.69 ^a^	13.64 ^e–i^	15.12 ^b–e^	14.38 ^abcd^
4	15.00 ^a^	9.11 ^fg^	12.06 ^a^	14.89 ^b–f^	16.35 ^ab^	15.62 ^ab^
5	14.67 ^ab^	4.01 ^ij^	9.34 ^a^	13.57 ^e–j^	14.53 ^b–g^	14.05 ^bcd^
6	15.11 ^a^	4.66 ^i^	9.88 ^a^	13.23 ^f–j^	15.89 ^abc^	14.56 ^abc^
7	15.48 ^a^	7.09 ^h^	11.29 ^a^	15.54 ^a–d^	17.10 ^a^	16.32 ^a^
8	14.03 ^abc^	3.51 ^ijk^	8.77 ^a^	13.44 ^e–j^	14.61 ^b–g^	14.03 ^bcd^
9	13.04 ^bcd^	1.20 ^l^	7.12 ^a^	13.38 ^e–j^	13.85 ^d–i^	13.62 ^bcde^
10	14.59 ^ab^	1.69 ^kl^	8.14 ^a^	12.62 ^h–k^	14.05 ^c–h^	13.34 ^cde^
11	12.86 ^bcd^	2.37 ^jkl^	7.62 ^a^	12.01 ^ijk^	12.78 ^g–k^	12.40 ^de^
12	15.60 ^a^	11.27 ^de^	13.43 ^a^	15.54 ^a–d^	17.06 ^a^	16.30 ^a^
13	12.25 ^cde^	1.09 ^l^	6.67 ^a^	11.18 ^k^	12.35 ^h–k^	11.77 ^e^
**Mean** ^**1**^	13.83 ^a^	4.46 ^b^		12.94 ^a^	14.38 ^b^	
**LSD**_**Stress**_	1.196				0.919	
**LSD**_**Treatment**_	10.574				2.108	
**LSD**_Stress*Treatment_	1.881				1.860	

Means of indices for three independent seedlings are shown. ^a–k^Different letters indicate statistically different mean values of Tukey’s HSD at *α* = 0.05.

1 One-or.

2 Two-Way ANOVA was used for the means separation. LSD denote the Least Significant Difference values calculated using the same method, either for single factors (Treatment; Stress) or their interaction.

* 1–13 numbers indicate active substances of seed treatments used, according to Table 1.

There was also a significant influence of the applied seed treatments on the level of transpiration (E; Table 5). Two-Way ANOVA indicated impact of the stress imposition, seed treatments, and combination of the factors on E during drought (each at *p* < 0.001, respectively). Whereas, the stress imposition and seed treatments after regeneration both reached the significance threshold of *p* < 0.001, respectively, and combination of the factors after regeneration reached *p* < 0.05. The use of all treatments under optimal irrigation conditions increased the level of transpiration in relation to plants whose seeds were not treated. The most intensive transpiration under optimal irrigation conditions was observed in plants whose seeds were treated using treatment No. 7, followed by No. 12 and 5; it was higher than the untreated control by 45%, 30%, and 28%, respectively. Under drought stress, the highest transpiration was recorded for plants treated with the treatment No. 12, exceeding the untreated control by 189% (Table 5).

**Table 5:** The influence of the seed treatment and drought stress on transpiration rate – E (mmol/m^2^ × s) during drought stress and after regeneration.

Treatment^*^	During drought stress^2^			After regeneration^2^		
	Control	Drought	Mean^1^	Control	Drought	Mean^1^
1	1.48 ^d^	0.36 ^hi^	0.92 ^a^	0.88 ^i^	1.34 ^fgh^	1.11 ^e^
2	1.64 ^bcd^	0.29 ^hi^	0.97 ^a^	1.04 ^hi^	1.40 ^e–h^	1.22 ^de^
3	1.88 ^abc^	0.80 ^efg^	1.34 ^a^	1.46 ^d–h^	1.87 ^a–d^	1.66 ^abc^
4	1.83 ^a–d^	0.88 ^ef^	1.36 ^a^	1.68 ^a–g^	1.95 ^abc^	1.81 ^ab^
5	1.89 ^ab^	0.45 ^ghi^	1.17 ^a^	1.51 ^c–g^	1.79 ^a–f^	1.65 ^abc^
6	1.72 ^bcd^	0.59 ^fgh^	1.16 ^a^	1.47 ^d–h^	1.90 ^a–d^	1.69 ^abc^
7	2.15 ^a^	0.76 ^efg^	1.45 ^a^	1.70 ^a–g^	1.97 ^ab^	1.83 ^ab^
8	1.79 ^a–d^	0.48 ^ghi^	1.13 ^a^	1.47 ^d–h^	1.52 ^b–g^	1.50 ^bcde^
9	1.49 ^cd^	0.21 ^hi^	0.85 ^a^	1.59 ^b–g^	1.60 ^b–g^	1.59 ^abcd^
10	1.67 ^bcd^	0.10 ^i^	0.88 ^a^	1.90 ^a–d^	1.74 ^a–f^	1.82 ^bcd^
11	1.77 ^a–d^	0.35 ^hi^	1.06 ^a^	1.45 ^d–h^	1.58 ^b–g^	1.51 ^bcd^
12	1.92 ^ab^	1.04 ^e^	1.48 ^a^	1.81 ^a–e^	2.06 ^a^	1.94 ^a^
13	1.68 ^bcd^	0.22 ^hi^	0.95 ^a^	1.25 ^ghi^	1.38 ^e–h^	1.32 ^cde^
**Mean** ^**1**^	1.76 ^a^	0.50 ^b^		1.48 ^a^	1.70 ^b^	
**LSD**_**Stress**_	0.116			0.129		
**LSD**_**Treatment**_	1.400			0.400		
**LSD**_Stress*Treatment_	0.387			0.452		

Means of indices for three independent seedlings are shown. ^a–i^Different letters indicate statistically different mean values of Tukey’s HSD at *α* = 0.05.

1 One-or.

2 Two-Way ANOVA was used for the means separation. LSD denote the Least Significant Difference values calculated using the same method, either for single factors (Treatment; Stress) or their interaction.

* 1–13 numbers indicate active substances of seed treatments used, according to Table 1.

The first and fundamental physiological response of plants to drought stress is a decrease in photosynthetic efficiency because of closing the stomata. Maintaining the balance between CO_2_ exchange and transpiration is necessary to maximize the CO_2_ assimilation in photosynthesis and, at the same time, to reduce water loss (Kollist et al., 2019). In our previous study of the use of seed treatments in barley plants under drought stress (Radzikowska et al., 2020), the highest increase in CO_2_ assimilation was also noted in objects treated with SDHI. Whereas our research on barley showed a significant reduction in transpiration in objects under drought stress, in the case of maize, stressed plants retained open stomata and carried out transpiration, although at significantly lower levels than plants with optimal hydration. This is probably due to mechanisms of the water use efficiency, which is twofold higher in C4 than in C3 plants (Madhava Rao et al., 2006). The plants with SDHI seed treatments applied showed the highest levels of CO_2_ assimilation (Table 4), transpiration (Table 5), and RWC (Table 3). A linear relationship between the decrease in the efficiency of CO_2_ assimilation and RWC was postulated (Marshall and Dumbroff, 1999). Water balance in maize plants could depend on structural features such as a low cell wall elastic modulus, which allows to preserve transpiration while maintaining turgor.

The highest values of stomatal conductance (Gs) under optimal water regime were found in plants whose seeds were treated using treatment No. 7 (67% higher than the untreated control), followed by treatments No. 3, 4, 5, and 12 (higher than the untreated control by 44%; Table 6; Supplementary Table 2). On the other hand, during drought stress, the significantly highest stomatal conductance values were recorded for plants whose seeds were treated using treatment No. 12, followed by No. 3 and 4 (exceeding the untreated control by 200% and 150%, respectively). Analysis using One- and Two-Way ANOVA indicated that at both stages-during drought and after regeneration-both stress imposition and seed treatment were significant (*p* < 0.001). Whereas, the combination of factors reached the significance level of *p* < 0.001 during drought and was not significant after regeneration (*p* = 0.072).

**Table 6:** The influence of the seed treatment and drought stress on stomatal conductance Gs (mol/m^2^ × s) during drought stress and after regeneration.

Treatment^*^	During drought stress^2^			After regeneration^2^		
	Control	Drought	Mean^1^	Control	Drought	Mean^1^
1	0.09 ^e^	0.02 ^hi^	0.05 ^a^	0.04 ^f^	0.08 ^b–e^	0.06 ^c^
2	0.10 ^de^	0.01 ^i^	0.05 ^a^	0.06 ^ef^	0.07 ^def^	0.06 ^c^
3	0.13 ^abc^	0.05 ^fg^	0.09 ^a^	0.07 ^def^	0.11 ^ab^	0.09 ^abc^
4	0.13 ^abc^	0.05 ^fg^	0.09 ^a^	0.09 ^b–e^	0.11 ^ab^	0.10 ^ab^
5	0.13 ^abc^	0.02 ^hi^	0.07 ^a^	0.08 ^cde^	0.11 ^abc^	0.09 ^abc^
6	0.11 ^cd^	0.03 ^gh^	0.07 ^a^	0.07 ^c–f^	0.12 ^ab^	0.10 ^abc^
7	0.15 ^a^	0.04 ^gh^	0.09 ^a^	0.09 ^bcd^	0.12 ^ab^	0.10 ^ab^
8	0.12 ^bcd^	0.02 ^hi^	0.07 ^a^	0.07 ^c–f^	0.10 ^bcd^	0.09 ^abc^
9	0.09 ^e^	0.01 ^i^	0.05 ^a^	0.07 ^c–f^	0.09 ^b–e^	0.08 ^abc^
10	0.11 ^de^	0.02 ^hi^	0.06 ^a^	0.07 ^def^	0.09 ^bcd^	0.08 ^abc^
11	0.12 ^bcd^	0.04 ^g^	0.08 ^a^	0.07 ^c–f^	0.09 ^b–e^	0.08 ^bc^
12	0.13 ^ab^	0.06 ^f^	0.10 ^a^	0.10 ^bcd^	0.14 ^a^	0.12 ^a^
13	0.10 ^de^	0.01 ^i^	0.06 ^a^	0.06 ^ef^	0.07 ^def^	0.06 ^c^
**Mean** ^**1**^	0.11 ^a^	0.03 ^b^		0.07 ^a^	0.10 ^b^	
**LSD**_**Stress**_	0.008			0.009		
**LSD**_**Treatment**_	0.094			0.037		
**LSD**_Stress*Treatment_	0.019			0.034		

Means of indices for three independent seedlings are shown. ^a–i^Different letters indicate statistically different mean values of Tukey’s HSD at *α* = 0.05.

1 One-or.

2 Two-Way ANOVA was used for the means separation. LSD denote the Least Significant Difference values calculated using the same method, either for single factors (Treatment; Stress) or their interaction.

* 1–13 numbers indicate active substances of seed treatments used, according to Table 1.

Drought stress caused the strongest decrease in the intercellular CO_2_ concentration (Ci) in plants treated using seed treatments No. 3, 4, and 12 (Table 7). Data analysis using One- and Two-Way ANOVA indicated that stress imposition, seed treatments, and the interaction of both factors had the impacts on Ci during drought and after regeneration (*p* < 0.001).

**Table 7:** The influence of the seed treatment and drought stress on sub-stomatal CO_2_–Ci (μmol/mol) during drought stress and after regeneration.

Treatment^*^	During drought stress^2^			After regeneration^2^		
	Control	Drought	Mean^1^	Control	Drought	Mean^1^
1	116.7 ^k^	174.7 ^de^	145.7 ^ab^	79.0 ^i–l^	79.5 ^i–l^	79.3 ^ef^
2	116.7 ^k^	187.7 ^d^	152.2 ^ab^	65.3 ^l^	79.3 ^i–l^	72.3 ^f^
3	155.7 ^e–h^	58.5 ^m^	107.1 ^b^	89.3 ^g–j^	131.3 ^bcd^	110.3 ^bcd^
4	167.0 ^def^	56.0 ^m^	111.5 ^b^	128.3 ^bcd^	136.3 ^abc^	132.3 ^ab^
5	152.3 ^fgh^	165.7 ^efg^	159.0 ^ab^	88.0 ^g–j^	118.5 ^c–f^	103.3 ^bcde^
6	148.5 ^fgh^	145.7 ^ghi^	147.1 ^ab^	96.0 ^ghi^	130.0 ^bcd^	113.0 ^bcd^
7	169.0 ^def^	84.0 ^l^	126.5 ^b^	104.0 ^e–h^	133.3 ^bcd^	118.7 ^abc^
8	153.5 ^e–h^	138.0 ^hij^	145.8 ^ab^	73.7 ^jkl^	122.0 ^cde^	97.8 ^cdef^
9	121.3 ^jk^	305.7 ^b^	213.5 ^ab^	74.3 ^jkl^	100.3 ^fgh^	87.3 ^def^
10	124.0 ^jk^	460.0 ^a^	292.0 ^a^	116.7 ^def^	107.0 ^efg^	111.8 ^bcd^
11	125.7 ^ijk^	233.7 ^c^	179.7 ^ab^	67.0 ^kl^	85.0 ^h–k^	76.0 ^ef^
12	168.5 ^def^	46.2 ^m^	107.3 ^b^	153.7 ^a^	143.3 ^ab^	148.5 ^a^
13	106.0 ^k^	309.7 ^b^	207.8 ^ab^	66.3 ^kl^	76.7 ^jkl^	71.5 ^f^
**Mean** ^**1**^	140.4 ^b^	181.9 ^a^		92.4 ^b^	111.0 ^a^	
**LSD**_**Stress**_	38.14			11.50		
**LSD**_**Treatment**_	152.10			29.97		
**LSD**_Stress*Treatment_	21.01			19.12		

Means of indices for three independent seedlings are shown. ^a–m^Different letters indicate statistically different mean values of Tukey’s HSD at *α* = 0.05.

1 One-or.

2 Two-Way ANOVA was used for the means separation. LSD denote the Least Significant Difference values calculated using the same method, either for single factors (Treatment; Stress) or their interaction.

* 1–13 numbers indicate active substances of seed treatments used, according to Table 1.

**Figure 2:**
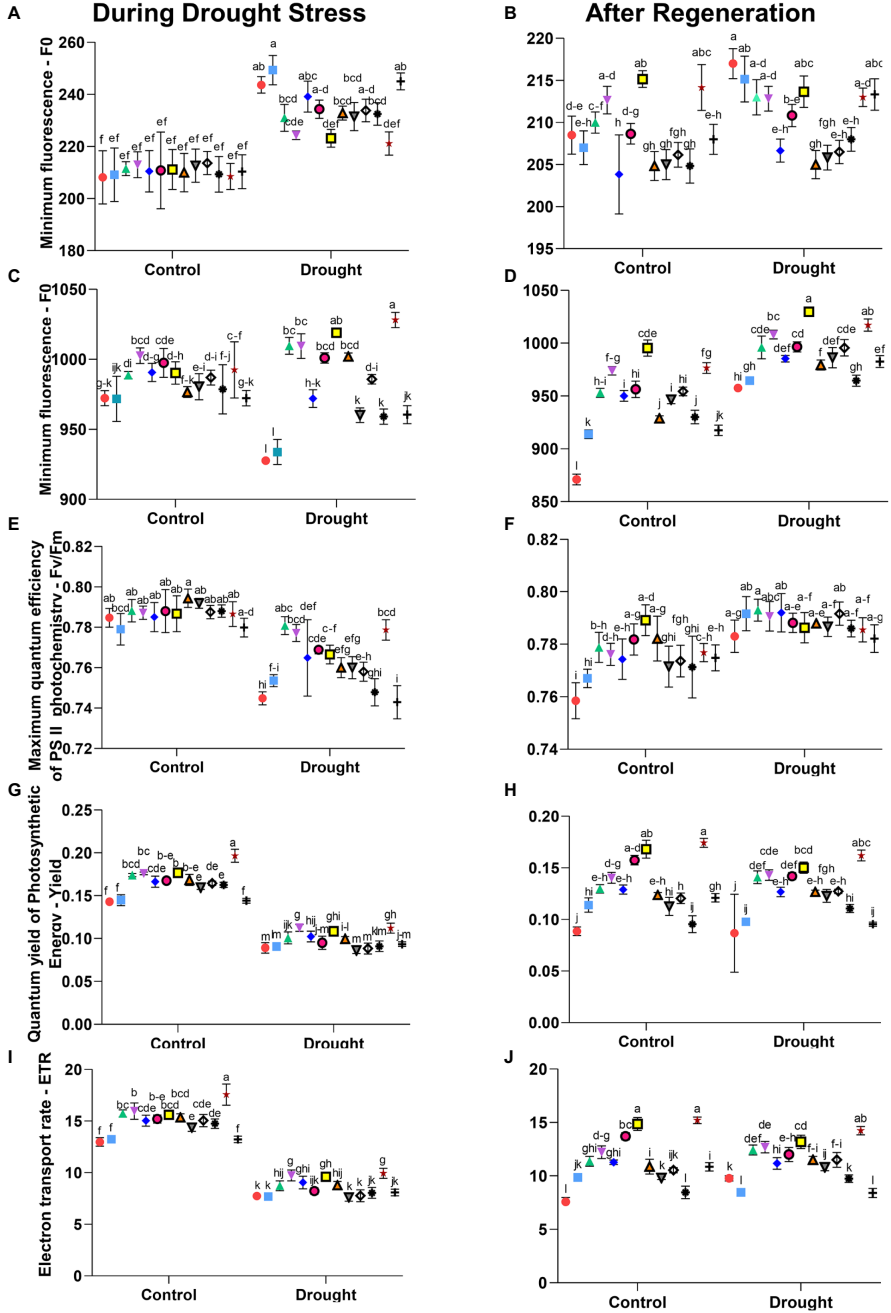
Parameters of chlorophyll fluorescence (non-nominated units): left column includes graphs with measurements during drought stress and right column the respective graphs with measurements after regeneration. Letters a-l indicate statistically different mean values (*α* = 0.05) of Tukey’s HSD. The values of Least Significant Difference calculated using the same method, as well as the means over single factor (treatment or stress), their significance in One-Way ANOVA and Tukey’s HSD are presented in Supplementary Table 4. Symbols indicate active substances of seed treatments used, according to Table 1: 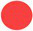
–1, 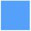
−2, 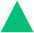
–3, 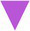
−4, 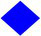
−5, 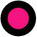
−6, 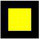
−7, 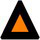
−8, 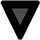
−9, 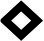
−10, 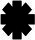
−11, 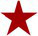
−12, 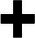
−13.

Studies of wheat under drought stress showed that the highest values of all measured photosynthetic parameters-CO_2_ assimilation, transpiration, stomatal conductance, and PSII efficiency-were obtained for a treatment in which a formulation from the SDHI group was used (Ajigboye et al., 2017). These results are confirmed in our research, not only in the above-described gas exchange parameters (Tables 4–7), but also in the investigated effectiveness of PSII measured as chlorophyll fluorescence (Figure 2; Supplementary Table 3). Furthermore, changes in the SDHI-treated plants may be under the genetic control, therefore such treatments may influence many aspects of photochemistry and plant physiology alike (Ajigboye et al., 2017). Overall, the significance of drought stress was comparable to that of seed treatments and the combination of both variables, both during drought and after regeneration (*p* < 0.001; Supplementary Table 4). The seed treatments had a comparably smaller significance for a few parameters (Fm during drought stress; Y and ETR after regeneration; Supplementary Table 4).

Based on the analysis of the chlorophyll fluorescence measurement results (Figure 2), we found a significant effect of the applied seed treatments on the following parameters measured after the plants’ adaptation to darkness: minimum fluorescence (F0), maximum fluorescence (Fm), maximum quantum efficiency of PSII photochemistry (Fv/Fm). The following parameters measured in the light were affected: quantum yield of photosynthetic energy (Yield) and the electron transport rate (ETR).

The highest values of minimum fluorescence (F0) during drought stress were found for plants with seeds treatments No. 2 and 13 and for the non-treated control (Figure 2A). On the other hand, the group with the lowest F0 values includes the plants with seeds treatments No. 7 and 12.

For plants treated with seeds treatment No. 4, and then No. 6 and 12, the significantly highest values of maximum fluorescence (Fm) under optimal irrigation conditions were found (Figure 2C). Under drought stress, the significantly highest Fm values were found for plants with seeds treatments No. 12, and then 7; these values were higher than for control plants with non-treated seeds by 11% and 10%, respectively. Decreased peak values of maximum fluorescence are usually caused by damage to the PSII preventing the complete reduction in electron acceptors. As previously demonstrated, the fungicide from the SDHI group increased the effectiveness of open reaction centers as early as 4 h after application (Ajigboye et al., 2014).

The values of the Fv/Fm parameter determining the maximum quantum efficiency of PSII photochemistry after adaptation to darkness did not show substantial differences among the treatments tested (Figure 2E). In plants with optimal hydration, the values of the Fv/Fm ranged from 0.7 to 0.8 and were similar to those recorded for most plant species in the absence of stressful conditions (Maxwell and Johnson, 2000). Under the drought stress, however, significantly the highest values of this parameter were found in plants with seeds treatments No. 3, 4, and 12, and the lowest in control plants with non-treated seeds and those with treatment No. 13. The differences in the decrease in the value of this parameter during drought stress were probably due to the differences in degradation rates of PSII reaction centers (van Wijk et al., 1994) and/or the different repair rates of the D1 protein (Nishiyama et al., 2006). The highest values of the quantum yield of photosynthetic energy (Yield), both under optimal irrigation conditions and under drought stress, were recorded for plants with seeds treatments No. 12, 7, and 4 (Figure 2G). Under optimal hydration conditions, the values of Yield exceeded those in control plants with non-treated seeds by 38%, 24%, and 23%, respectively, whereas under drought – by 26%, 27%, and 21%, respectively.

We also observed a significant influence of the applied seed treatments on the electron transport (ETR). The highest electron transport rate among plants grown under optimal hydration conditions was recorded for those with seeds treatment No. 12 (higher than control plants with non-treated seeds by 35%), whereas under drought, for plants with seeds treatments No. 12, 4, and 7 (higher than control plants with non-treated seeds by 29%, 26%, and 24%, respectively; Figure 2I). The Yield parameter determines the share of light quanta used effectively in photochemical transformations from the total number of absorbed quanta; whereas, the ETR parameter determines the electron transport rate and is dependent on Yield, since the transport of 1 electron by PSI and PSII requires the absorption of 2 PAR quanta (Genty et al., 1989). As observed in our previous work with barley (Radzikowska et al., 2020) and by others (Berdugo et al., 2012), the values of chlorophyll fluorescence parameters – F0, Fm, Y, and ETR – depended on the type of seed treatment used, and the use of treatments, especially those containing sedaxane and fluxapyroxad, had a positive effect on mitigating the damage to the photosynthetic apparatus caused by drought.

### Effect of the seed treatments on the physiological state of plants after regeneration

The assessment of the physiological condition of plants at regeneration that followed the previously induced drought stress, was carried out in a similar way to that during stress. The photosynthetic activity and the chlorophyll fluorescence were measured.

The significant influence of the applied seed treatments on the efficiency of photosynthesis measured after plant regeneration has been observed: During drought stress treatments alone were significant for all but Fm, whereas after regeneration – for all but ETR (Supplementary Table 4). The highest levels of CO_2_ assimilation (A) in previously drought-stressed plants were found in those whose seeds were treated with treatments No. 7, 12, and 4 (higher than the non-treated control by 53%, 53%, and 46%, respectively). In plants growing under optimal hydration conditions, during the measurement after regeneration, the highest values of CO_2_ assimilation were recorded for those whose seeds were treated treatments No. 12 and 7. The lowest levels of photosynthesis efficiency in plants previously drought-stressed were found for the untreated control and for those with seeds treatments No. 2, 13, and 11 (Table 4).

The highest level of transpiration (E) measured after regeneration was found in plants previously drought-stressed with seeds treatment No. 12, followed by 7 and 4, and the increase in the E value in relation to the non-treated control was 54%, 47%, and 45%, respectively. Similarly, under optimal irrigation conditions, the group of plants with the most intensive transpiration (E) included these with seeds treatments No. 10, 12, 7, and 4. The lowest water transpiration (E) from the leaves, among the previously drought-stressed plants, was in the non-treated control and in those with seeds treatments No. 13 and 2 (Table 5). The analysis of gene expression, chlorophyll gas exchange, and fluorescence measurements, similar to our studies for barley (Radzikowska et al., 2020) and maize, showed that the SDHI fungicides increased the efficiency of photosynthesis and of PSII under drought stress (Ajigboye et al., 2017).

The highest stomatal conductance (Gs) measured after regeneration, both under optimal hydration conditions and after the drought, was found in plants with seeds treatment No. 12 (higher than control plants with non-treated seeds by 150% and 75%, respectively; Table 6). In the same objects, the significantly highest level of intercellular CO_2_ concentration (Ci) was found, which exceeded the non-treated control in plants previously stressed with drought by 80% (Table 7). On the other hand, the significantly lowest values of both of these photosynthesis parameters in plants previously subjected to stress were recorded for those that were not treated, and for plants with seeds treatments No 13 and 2. A decrease in Ci is a reaction characteristic for maize under drought stress (Lal and Edwards, 1996). At the same time, high values of intercellular CO_2_ concentration and of stomatal conductance in SDHI-treated plants noted during regeneration may result from the fact that these plants have not lost the ability to transpire. Increased Ci could be an artifact due to patchy stomatal closure (Sharkey et al., 1990).

Measurements of chlorophyll fluorescence after regeneration indicated that all applied seeds treatments had positive effects on the regeneration process of PSII. Based on the measurements after regeneration in the group of previously drought-stressed plants, the highest values of the minimum fluorescence (F0) were observed for the control plants with non-treated seeds, and for the plants with seeds treatment No. 2 (Figure 2B). Measurements of the maximum fluorescence (Fm) after plant regeneration showed that the highest values of Fm in the group of plants previously stressed with drought were observed in those with seeds treatments No. 7, 12, and 4 (exceeding the non-treated control by 7%, 6%, and 5%, respectively), and were the lowest in the non-treated control plants (Figure 2D).

The analysis of maximum quantum efficiency of PSII photochemistry (Fv/Fm) after regeneration showed significant increases compared to the respective values under drought, although the differences between Fv/Fm parameter after regeneration between treatments were small (Figure 2F). The differences in the values of this parameter during drought and their absence after regeneration confirm the earlier assumption that specific treatments used in our study influenced the repair rate of PSII and D1 protein reaction centers. It would be worthwhile to carry out more measurement cycles in the future to precisely estimate the rate of recovery. Such estimates, when compared across varieties, may be used as proxy for drought tolerance in maize and aid the choice of seeds to be grown under given climatic constraints.

Both parameters of chlorophyll fluorescence measured in the light-Yield and ETR-indicated the highest efficiency of PSII after regeneration in plants with seeds treatments No. 12 and 7, both in the group of plants previously stressed by drought and those grown under optimal hydration conditions. The quantum yield of photosynthetic energy (Yield) in the plants previously stressed by drought and with seeds treatments No. 12 and 7 exceeded those in the control non-treated plants by 86% and 72%, respectively (Figure 2H), and the electron transport rate (ETR) by 46% and 35%, respectively (Figure 2J).

The results of the RWC, gas exchange, and chlorophyll fluorescence measurements were generally consistent with previous studies of six maize varieties tolerant and susceptible to drought (Cruz de Carvalho et al., 2011). Assuming that SDHI fungicides can cause a change in the expression of genes in plants (Shkliarevskyi et al., 2019), their application allowed to maintain turgor and photosynthetic activity during drought and accelerated regeneration, and therefore increased the tolerance of maize to drought.

### Principal components analysis

**Figure 3:**
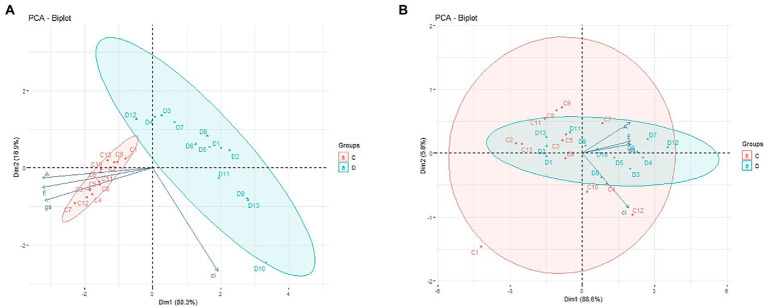
PCA of plant photosynthesis parameters. Projection of the variables on the component plane (1 × 2): **(A)**—during drought stress; **(B)**—after plant regeneration. C, control (well-watered); D, drought stress; 1–13: seed treatments (Table 1). Each of the vectors represents one variable, and their sizes and directions describe the effects they have on the major components.

To assess the relationships among the applied seed treatments 1 through 13 and the analyzed parameters of plant photosynthesis (Ci; Gs; A; E) in the period of drought (Figure 3A) and after plant regeneration (Figure 3B), principal components analysis (PCA) was performed. PCA analysis allows to identify the variables that have the greatest impact on the individual principal components and facilitates the interpretation of the impact of the applied seed treatments on drought tolerance of maize, which in turn may have an impact on the yield. Based on this analysis, the plants treated with seed treatments from the SDHI group (No. 12, 7, 6, 4, and 3) showed significantly higher tolerance to drought, showing significantly higher CO_2_ assimilation (A) (Figure 3A). Seed treatments No. 12, 4, 3, and 7 also improved plant growth under optimal irrigation conditions. Similarly, plants with those seeds treatments showed significantly higher values of transpiration (E) and stomatal conductance (Gs), which proves that they fared better under drought (Tables 5, 6). Moreover, after the stress factor subsided (regeneration; Figure 3B), plants with seeds treatments No. 12, 7, 4, and 3 returned to better physiological conditions faster (data not shown). Thus, we confirmed the beneficial effect of mortars from the SDHI group on the functioning of plants under drought, and under changing growth conditions (alternating drought and irrigation).

## Conclusions and outlook

The seed treatments used in this study varied greatly in their influence on the maize germination process and the physiological state of seedlings under drought and after regeneration. The use of seed treatments based on substances from the SDHI group (sedaxane) contributed to the improvement of the development of the maize root system. Furthermore, these treatments boosted the functioning of plants growing under optimal soil moisture conditions and under drought stress, influencing both the photosynthesis process, increasing the absorption of CO_2_ in relation to non-treated controls, and improving the parameters of chlorophyll fluorescence. The obtained results indicate the possibility of using substances from the SDHI group to reduce the stress of drought in maize cultivation, especially in the face of progressing climate change.

## Data availability statement

The original contributions presented in the study are included in the article/Supplementary material, further inquiries can be directed to the corresponding authors.

## Author contributions

DR: conceptualization, methodology, investigation, writing–original draft, writing–review and editing, visualization, and project administration. PŁK: investigation, writing–original draft, and writing–review and editing. MG: investigation. RG-W: visualization. MN: writing–review and editing. ZS: conceptualization, methodology, investigation, writing–original draft, and writing–review and editing. All authors contributed to the article and approved the submitted version.

## Funding

This research did not receive any specific funding. Funding for open access to this research was provided in part by University of Tennessee’s Open Publishing Support Fund. The publication was co-financed within the framework of Polish Ministry of Science and Higher Education programme as “Regional Initiative Excellence” in years 2019–2022, project no. 005/RID/2018/19.

## Conflict of interest

The authors declare that the research was conducted in the absence of any commercial or financial relationships that could be construed as a potential conflict of interest.

## Publisher’s note

All claims expressed in this article are solely those of the authors and do not necessarily represent those of their affiliated organizations, or those of the publisher, the editors and the reviewers. Any product that may be evaluated in this article, or claim that may be made by its manufacturer, is not guaranteed or endorsed by the publisher.

## Supplementary material

SUPPLEMENTARY TABLE 1 Data for Relative Water Content.

SUPPLEMENTARY TABLE 2 Data for photosynthesis related measurements.

SUPPLEMENTARY TABLE 3 Data for fluorescence related parameters.

SUPPLEMENTARY TABLE 4 The values of Least Significant Difference calculated using Tukey’s HSD values (*α* = 0.05) after Two-Way ANOVA for the parameters of chlorophyll fluorescence (Supplementary Table 3).
